# What is the quality of clinical practice guidelines for the treatment of acute lateral ankle ligament sprains in adults? A systematic review

**DOI:** 10.1186/s12891-019-2750-6

**Published:** 2019-08-31

**Authors:** Toni Green, Grant Willson, Donna Martin, Kieran Fallon

**Affiliations:** 1 ANU Medical School, College of Health and Medicine, Australian National University, ACT, Acton, Australia; 2 Discipline of Physiotherapy, University of Canberra, ACT, Bruce, 2617 Australia; 3 Elite Rehab and Sports Physiotherapy, Deakin, Canberra, Australia

**Keywords:** Physiotherapy, Physical Therapy/Rehabilitation, Physical Therapy, Modalities, Non-Steroidal Anti-Inflammatory Drugs (NSAIDs)

## Abstract

**Background:**

Acute lateral ankle ligament sprains (LALS) are a common injury seen by many different clinicians. Knowledge translation advocates that clinicians use Clinical Practice Guidelines (CPGs) to aid clinical decision making and apply evidence-based treatment. The quality and consistency of recommendations from these CPGs are currently unknown. The aims of this systematic review are to find and critically appraise CPGs for the acute treatment of LALS in adults.

**Methods:**

Several medical databases were searched. Two authors independently applied inclusion and exclusion criteria. The content of each CPG was critically appraised independently, by three authors, using the Appraisal of Guidelines for REsearch and Evaluation (AGREE II) instrument online version called My AGREE PLUS. Data related to recommendations for the treatment of acute LALS were abstracted independently by two reviewers.

**Results:**

This study found CPGs for physicians and physical therapists (Netherlands), physical therapists, athletic trainers, physicians, and nurses (USA) and nurses (Canada and Australia). Seven CPGs underwent a full AGREE II critical appraisal. None of the CPGs scored highly in all domains. The lowest domain score was for domain 5, applicability (discussion of facilitators and barriers to application, provides advice for practical use, consideration of resource implications, and monitoring/auditing criteria) achieving an exceptionally low joint total score of 9% for all CPGs. The five most recent CPGs scored a zero for applicability. Other areas of weakness were in rigour of development and editorial independence.

**Conclusions:**

The overall quality of the existing LALS CPGs is poor and majority are out of date. The interpretation of the evidence between the CPG development groups is clearly not consistent. Lack of consistent methodology of CPGs is a barrier to implementation.

**Systematic review:**

Systematic review registered with PROSPERO (CRD42015025478).

**Electronic supplementary material:**

The online version of this article (10.1186/s12891-019-2750-6) contains supplementary material, which is available to authorized users.

## Background

Lateral ankle ligament sprains (LALS) are common [1, 2] and costly [3] soft tissue injuries. An acute LALS is defined as “an acute traumatic injury to the lateral ligament complex of the ankle joint as a result of excessive inversion of the rear foot or a combined plantarflexion and adduction stress to the foot. This injury usually results in initial deficits of function and disability” [4]. This definition of an acute LALS has been endorsed by the International Ankle Consortium [5–7]. The acute phase is usually defined as less than two weeks after the injury [8]. This acute phase corresponds to the first phase of biological ligament healing known as the inflammatory phase [9].

LALS are a common occurrence in the general population, indoor sports [10], field athletes [11], military personnel [12] and dancers [13, 14]. Acute phase signs and symptoms are often weakness, stiffness, pain and swelling. Generally these resolve within six weeks but may persist for years [15]. A history of LALS may predispose to reduced movement such as ankle dorsiflexion [16]. Complications from LALS are also costly and include chronic ankle instability (CAI) [16, 17], post traumatic ankle osteoarthritis (PTOA) [18] and an increased fall risk in older populations [19]. CAI is defined as “an encompassing term used to classify a subject with both mechanical and functional instability of the ankle joint. To be classified as having chronic ankle instability, residual symptoms (“giving way” and feelings of ankle joint instability) should be present for a minimum of one year post-initial sprain” [4]. It is usual for a CAI to be under the care of a medical practitioner and/or physical therapist over an extended time, sometimes years.

An acute LALS may be seen by many different people some of whom are not experienced highly trained clinicians. This is suboptimal as the condition requires correct diagnosis and if necessary, a referral. Acute LALS injuries may present to first aid officers at the workplace or at sporting events, to emergency departments [20, 21] or to a variety of primary contact clinicians. Sports physicians, physical therapists, athletic trainers, nurse practitioners, school nurses, doctors in general practice, accident and emergency staff (doctors, nurses and physical therapist), community pharmacists (pharmacists, pharmacy assistants and shop assistants) and first aid officers [22] may diagnose, advise, refer or offer treatments for acute LALS based on their prior education, training, continuing professional development and CPGs.

Knowledge translation has been described by Lang as “any activity or process that facilitates the transfer of high quality evidence from research into effective changes in health policy, clinical practice or products” [23]. It involves research, education, quality improvement and electronic systems [23]. CPGs are used to encourage knowledge translation of evidence based medicine [24] to the clinical setting. The development of CPGs is constantly improving and assistance in developing CPGs can be found by reference to such instruments as the Appraisal of Guidelines for REsearch & Evaluation II (AGREE II) [25] .

## Objectives

The primary aim of this systematic review is to find and critically appraise all CPGs related to the treatment of acute LALS in adults. The secondary aim is to determine if CPGs are using the same studies to support their treatment recommendations for acute LALS.

## Methods

### Protocol and registration

The search strategy, inclusion and exclusion criteria were specified and documented in advance and registered with PROSPERO (registration number: CRD42015025478).

### Database search strategy

An electronic search was conducted across medical literature databases. All searches were performed in October 2017. The Cochrane Library, MEDLINE, CINAHL, Sportdiscus, Web of Science, Scopus, USA National Guideline Clearinghouse and PEDro databases were searched to find all CPGs for treatment of LALS. The search strategy was agreed upon after discussion with an experienced librarian and was refined through team discussion. The search strategy of medical databases used medical subject headings and free text search terms (mesh). Specifically, terms of (“ankle injur*” OR “ankle sprain*” OR “sprained ankle*”) and guideline* were used. In addition, other sources such as Google Scholar, handsearching and personal communication supplied guidelines. Only CPGs published in English were retrieved. See Additional file 1: Search Strategy and List of Articles, excel spreadsheet for more details.

### Study selection

Records were imported into referencing software (Endnote X7, Thomson Reuters, New York, New York USA) and all duplicates were removed using this software.

### Inclusion criteria

The review considered CPGs for adults 18 years and older with LALS and acute treatment recommendations.

### Exclusion criteria

The review did not consider CPGs for adults 18 years and older with diagnoses of ankle fractures or syndesmosis ankle sprains. The authors are aware that distal fibular avulsion fractures are common in LALS and are managed as LALS.

### Data collection and risk of Bias

Study inclusion was determined by 2 authors (TG, GW) who independently considered title, abstract and full text. There was an absolute rate of agreement between the two reviewers of 94% and a prevalence-adjusted and bias-adjusted kappa [26] of 0.88 (95% CI 0.68 to 0.98). Disagreement was resolved during discussion with another author (KF).

### AGREE II data collection process

The Appraisal of Guideline Research and Evaluation II (AGREE II) [25] is a standardised and internationally recognised CPG critical appraisal tool. It was developed to address the variable quality of CPGs by supplying a structured process to evaluate the methodological rigour and transparency of CPG development and quality of reporting of CPG development. The AGREE II consists of 23 items, which are grouped into six domains: scope and purpose (3 items), stakeholder involvement (3 items), rigour of development (8 items), clarity of presentation (3 items), applicability (4 items), and editorial independence (2 items). Each of these items is rated on a seven-point scale, ranging from 1 = strongly disagree to 7 = strongly agree. In addition, the two final items provide the appraiser with the opportunity to make an overall judgement of the CPG. The appraisers rate the overall quality of the guideline on a seven-point scale ranging from 1 = lowest possible quality to 7 = highest possible quality. The appraiser can also respond to the question “I would recommend this guideline for use” by selecting the most appropriate response choice from “yes,” “yes with modifications” or “no.” Domain scores are calculated by calculating the sum of all the scores of the individual items in a domain and then by scaling the total as a percentage of the maximum possible score for that domain. The My AGREE PLUS is the online software version of the tool and appraisers are sent hyperlinks to the group appraisal site via email. The appraisers appraise the guideline online using this software. The My AGREE PLUS calculates a score out of 100 for each domain for each guideline.

The AGREE II [25] has undergone both validity [27] and reliability testing [28] and results have been published in peer-reviewed journals. These results have shown the AGREE II to be a valid and reliable instrument, with sufficient inter-rater reliability. The AGREE II tool is supported by two systematic reviews that found that it is the only validated tool for assessment of CPG and, in addition, it enables production of a numerical score for the critically appraised CPG [29, 30]. AGREE II recommends that at least two and preferably four appraisers rate a single practice guideline to increase the reliability of the assessment.

Three authors (TG, DM, KF) read the AGREE II user manual and watched the online tutorials from the AGREE II website (http://www.agreetrust.org/agree-ii/). All CPGs were reviewed independently, and conflicts were resolved by discussion until consensus was reached between all 3 authors. This was consistent with the methodology of a systematic review of osteoarthritis guidelines [31] and a systematic review of low back pain guidelines [32]*.*

### Recommendations for treatment of acute LALS data collection process

A data abstraction form in Microsoft Excel (2016)) was developed, piloted, and changed, as necessary. Data was later abstracted by one reviewer (TG) and verified independently by another reviewer (GW). Recommendations for the treatment of acute LALS were abstracted. Recommendations from CPGs about the use of specific treatment options were dichotomized to “recommended” or “not recommended”. In addition, a descriptive synthesis of the recommendations and their supporting evidence was undertaken. It was assessed if the same evidence had been used during formulation of the CPGs.

## Results

### Study selection

**Fig. 1:**
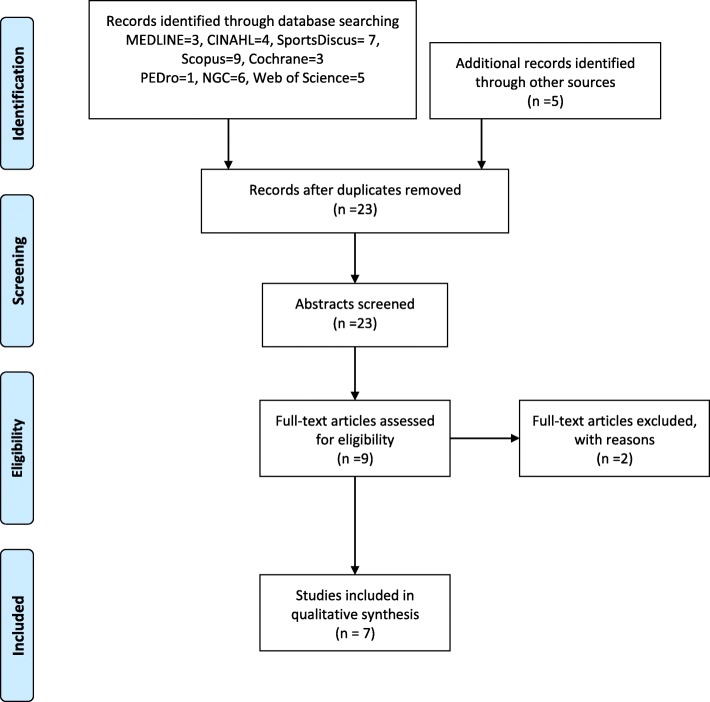
Results of the search strategy for international guidelines that have recommendations for acute management of lateral ankle ligament sprains. SR CPGS LALS PRISMA Flow Diagram

Forty-three articles were exported to Endnote X7 [33]. Twenty duplicates were removed. Twenty-three articles were independently reviewed. Initially nine CPGs were considered suitable. Subsequently, two CPGs were considered not suitable after discussion and agreement by authors (TG, GW, KF), namely a CPG for return to play after an LALS [34] and a syndesmosis injury CPG [35]. Seven CPGs were suitable for review using AGREE II see Fig. 1: Results of the search strategy for international guidelines that have recommendations for acute management of LALS and Table 1 for details of the included CPGs.

**Table 1:** Description of the lateral ankle ligament sprain clinical practice guidelines

Number	Publication	Name	Author	Target Health Professional
1	2006	Health care guideline: ankle sprain.	Fongemie, A., et al. [36]	Physicians and nurses from Institute for Clinical Systems Improvement. www. ICSI. Org Zugriff am
2	2006	KNGF guideline for physical therapy in patients with acute ankle sprain-practice guidelines.	Wees, P., et al. [37]	Physical therapists who are members of the Royal Dutch Society of Physical Therapy
3	2009	Occupational Health Nurse Practitioner (OHNP) Clinical Practice Guideline (CPG), Ankle/Foot Injury.	Fonceca [38]	Occupational health nurse practitioner from Carepoint Industrial Health Services
4	2011	Adult Care, Chapter 7, Musculoskeletal System.	Health Canada [39]	Nurses employed by Health Canada First Nations and Inuit Health Branch (FNIHB) CPG for Nurses in Primary Care
5	2012	Diagnosis, treatment, and prevention of ankle sprains: an evidence-based clinical guideline.	Kerkhoffs, G. M., et al. [40]	Physical therapists, orthopaedic and trauma surgeons, family, rehabilitation, occupational, and sports physicians, radiologists, and professionals involved in sport massage
6	2013	National Athletic Trainers’ Association position statement: conservative management and prevention of ankle sprains in athletes.	Kaminski, T. W., et al. [41]	Athletic trainers who are members of the American Athletic Trainers’ Association
7	2013	Ankle stability and movement coordination impairments: ankle ligament sprains: CPG linked to the international classification of functioning, disability and health from the orthopaedic section of the American Physical Therapy Association.	Martin, R. L., et al. [42]	Physical therapists who are members of the American Physical Therapy Association

Two CPGs were written for nurses [38, 39], one CPG for American athletic trainers [41], two CPGs for Dutch physical therapists and physicians [37, 40], one CPG for American physical therapists [42], and one organisational CPG for physicians and nurses [36]. The Canadian nurse CPG was web based [39].

### AGREE II analysis

**Fig. 2:**
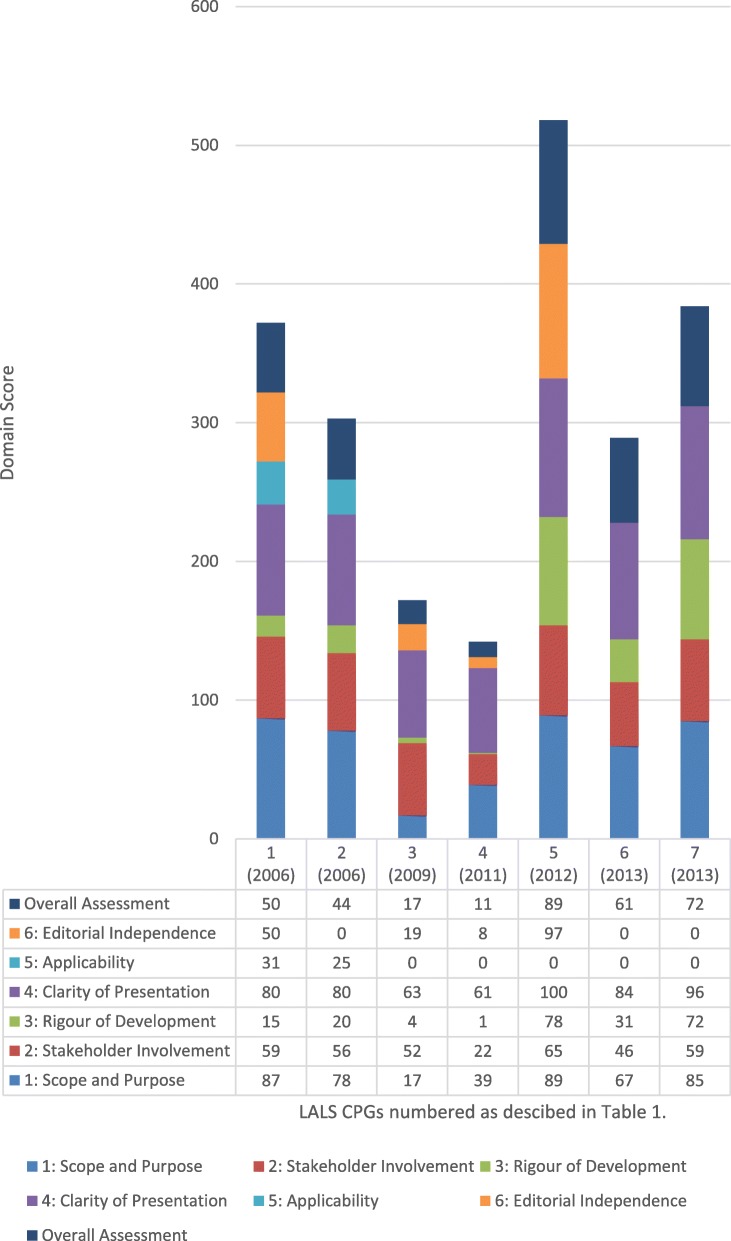
AGREE II results of lateral ankle ligament sprains clinical practice guidelines. AGREE II results of LALS CPGs

The AGREE II scores, which were derived from the three independent reviewers’ scores as a percentage of the maximum possible score, are shown in Fig. 2. The highest domain scores were for domain 4: clarity of presentation with all CPGs scoring above 61%. The second highest domain scores were for domain 1: scope and purpose with five CPGs scoring above 67%. The 2012 Dutch CPG [40] was the only CPG to receive 100% score in any domain (domain 4).

The lowest overall domain score was for domain 5: applicability (discussion of facilitators and barriers to application, provides advice for practical use, consideration of resource implications, and monitoring/auditing criteria) scoring a joint total score of 9% for all CPGs. The five most recent CPGs scored a zero for applicability. The second lowest domain score was domain 6: editorial independence. Three CPGs scored zero for this domain [37, 41, 42].

### Descriptive synthesis of studies for recommendations

For each of the appraised CPGs, the Level of Evidence (LOE) and Strength of Recommendation (SOR) grading scales used in the formulation of the respective recommendations were extracted from the guideline and tabulated (Table 2 Methods used to assess the quality of evidence to support the recommendations). Two CPGs [38, 39] had not reported the scale/category/LOE/SOR for their recommendations for treatment of LALS.

**Table 2:** Methods used to assess the quality of evidence to support the recommendations

Guideline 1, 2006 Evidence Grading System	
A. Primary Reports of New Data Collection:	
Class A: Randomized, controlled trial	
Class B: Cohort study	
Class C: Non-randomized trial with concurrent or historical controls, Case-control study, Study of sensitivity and specificity of a diagnostic test, Population-based descriptive study	
Class D: Cross-sectional study, Case series, Case report	
B. Reports that Synthesize or Reflect upon Collections of Primary Reports:	
Class M: Meta-analysis, Systematic review, Decision analysis, Cost-effectiveness analysis	
Class R: Consensus statement, Consensus report, Narrative review	
Class X: Medical opinion	
Guideline 2, 2006 LEVELS OF EVIDENCE	
1 one systematic review (A1 quality; see below) or at least two independent studies of A2 quality;	
2 at least two independent studies of B quality	
3 one study of A2 or B quality, or several studies of C quality;	
4 expert opinion, e.g. that of members of the Guideline Committee	
Quality levels (intervention and prevention)	
A1 Systematic reviews including at least some studies of A2 quality, with results consistent across individual studies.	
A2 Randomized comparative clinical trial (RCT) of sound methodological quality (randomized double-blind controlled trial) of sufficient size and consistency.	
B Randomized comparative clinical trial (RCT) of moderate quality or insufficient size; other comparative study (non-randomized comparative cohort study or case-control study).	
C Non-comparative study.	
D Expert opinion, e.g. that of members of the Guideline Committee.	
Guideline 3, 2009 none said	
Guideline 4, 2011 none said	
Guideline 5, 2012 Classification of methodological quality of individual studies	
A1 Systematic review of at least two independently conducted studies of A2 level	
A2 Randomised double-blind comparative clinical research of good quality of sufficient size Research relative to a reference test (a ‘golden standard’) with predefined cut-off points and independent assessment of the results of a test and golden standard, on a sufficiently large series of consecutive patients who all have had the index and reference test Prospective cohort study of sufficient size and follow-up, at which adequately controlled for ‘confounding’ and selective follow-up sufficient is excluded.	
B Comparative research, but not with all the features as mentioned under A2 (this includes patient–control research, cohort study) Research relative to a reference test, but not with all the attributes that are listed under A2 Prospective cohort study, but not with all the features as mentioned under A2 or retrospective cohort study or patient-monitoring research	
C Not comparative research	
D Opinion of experts	
Conclusions based on	
1 Research of level A1 or at least two examinations of level A2 performed independently of each other, with consistent results	
2 One examination of level A2 or at least two examinations of level B, performed independently of each other	
3 One examination of level B or C	
4 Opinion of experts	
Guideline 6, 2013	
The taxonomy includes ratings of A, B, or C for the strength of recommendation for a body of evidence. A being consistent and good quality patient-oriented evidence. B being inconsistent and limited quality patient-oriented evidence. C based on consensus, usual practice, opinion, disease-oriented or case series for studies of diagnosis, treatment, prevention, or screening. They recommendations were graded according to the Strength of Recommendation Taxonomy	
Guideline 7, 2013 LEVELS OF EVIDENCE	
I Evidence obtained from high-quality diagnostic studies, prospective studies, or randomized controlled trials	
II Evidence obtained from lesser-quality diagnostic studies, prospective studies, or randomized controlled trials (e.g., weaker diagnostic criteria and reference standards, improper, randomization, no blinding, less than 80% follow-up)	
III Case-control studies or retrospective studies	
IV Case series	
V Expert opinion	
GRADES OF RECOMMENDATION BASED ON STRENGTH OF EVIDENCE	
A Strong evidence A preponderance of level I and/or level II studies support the recommendation. This must include at least 1 level I study	
B Moderate evidence. A single high-quality randomized controlled trial or a preponderance of level II studies support the recommendation	
C Weak evidence A single level II study or a preponderance of level III and IV studies,s including statements of consensus by content experts, support the recommendation	
D Conflicting evidence Higher-quality studies conducted on this topic disagree with respect to their conclusions. The recommendation is based on these conflicting studies	
E Theoretical/ foundational evidence A preponderance of evidence from animal or cadaver studies, from conceptual models/principles, or from basic science/bench research supports this conclusion	
F Expert opinion Best practice based on the clinical experience of the guideline’s development team	

This study’s secondary aim was to determine if CPGs were using the same research studies to support their recommendations for treatments in the acute LALS phase. To that end the acute treatments and the supporting research cited for the specific recommended acute treatment in the CPGs were tabulated and organised according to the CPG’s age, with 1 showing the oldest CPG and 7 being the most recent (Table 3). Any sections that were blank in Table 3 shows that this acute treatment choice was not included in the CPG. All guidelines recommended progressive weightbearing with support. Ice was also recommended by all guidelines. Heat was not recommended by three CPGs. Ultrasound was not recommended by four guidelines [37, 40–42]. Conflicting recommendations occurred in four recommendations: graded joint mobilisations or mobilisation with movement, pulsating short wave diathermy, electrotherapy, and low-level laser. Thirty-one recommendations of the seventy-two were made without any studies cited to support this decision. These recommendations were decided upon by expert opinion or consensus.

**Table 3:** Supporting evidence in each of the seven clinical practice guidelines for acute treatment of lateral ankle ligament sprains

Acute Treatment	1 [36] (2006)	2 [37] (2006)	3 [38] (2009)	4 [39] (2011)	5 [40] (2012)	6 [41] (2013)	7 [42] (2013)
Progressive weight bearing with support depending on severity (tape, brace, boot, casting)	R [43–45]	R [45, 46]	R	R	R [45–48]	R [45, 47, 49]	R [47, 48, 50-56]
Ice	R	R [57]	R	R	R [57, 58]	R [57-61]	R [57, 58]
Compression	R	R	R	R	R [62–64]	R [65]	
Elevation	R	R	R	R	R	R	
Progressive strengthening exercises	R	R		R	R [66–70]	R [45, 49, 66, 71]	R [66–68, 72, 73]
Balance exercises	R	R		R	R [66–69]	R [74–76]	R [74–77]
NSAIDs/paracetamol	R# [78–83]		R	R		R [69, 84, 85]	
Refer on to another discipline			R	R	R		
Advice DVT risk with immobilisation	R				R		
Foot circle exercises	R			R			
Alphabet exercises				R			
Lymphatic drainage/soft tissue mobilisations							R [86]
Graded joint mobilisations or mobilisation with movement		R [73]			X [69, 73, 87]	R [88, 89]	R [44, 73, 90]
Pulsating short wave diathermy		X [91, 92]			X [91, 92]		R [92]
Electrotherapy		X			X [93-97]	R [98, 99]	R [97, 100]
Low-level laser		X			X [101]		R [101, 102]
Heat	X			X		X [103]	
Ultrasound		X [104]			X [105]	X [104]	X [104, 105]

Key: R = CPG recommends treatment, X = CPG does not recommend treatment, # = analgesic dose. Blank = this acute treatment choice was not included in CPG.

The studies that were cited in three or more of the CPGs were noted (Table 4). Five of these common studies were systematic reviews. The most cited systematic reviews were cited by four guidelines.

**Table 4:** Studies common in three or more clinical practice guidelines

Studies	Type	1 [36] (2006)	2 [37] (2006)	3 [38] (2009)	4 [39] (2011)	5 [40] (2012)	6 [41] (2013)	7 [42] (2013)
Pasila (1978) [92]	RCT		✓			✓		✓
Van der Windt (2002) [104]	SR		✓				✓	✓
Kerkhoff (2002) [45]	SR	✓	✓			✓	✓	
Bleakley (2004) [57]	SR		✓			✓	✓	✓
Bleakley (2006) [58]	RCT					✓	✓	✓
Van der Wees (2006) [73]	SR		✓			✓		✓
Kerkhoff (2007) [48]	SR					✓	✓	✓
Lamb (2009) [47]	RCT					✓	✓	✓
Bleakley (2010) [66]	RCT					✓	✓	✓

KEY: ✓ cited by this CPG, SR = Systematic Review, RCT = Randomised Clinical Trial

## Discussion

The primary aim of this systematic review is to identify and critically appraise evidence based CPGs for the acute treatment of LALS in adults. Two CPGs were written for nurses [38, 39], one CPG for American athletic trainers [41], two Dutch CPGs [37, 40], one CPG for American physical therapists [42], and one organisational CPG for physicians and nurses [36]. The more recent Dutch CPG is a multidisciplinary guideline who targets all care providers of LALS [40]. Considering that these are such common [1, 2] and potentially costly [3] soft tissue injuries it is surprising that so few CPGs exist for LALS.

It is also surprising that no published CPGs exist for community pharmacies (pharmacists, pharmacy assistants and shop assistants) or first aid officers. It is common for acute LALS patients to seek free advice at pharmacies. First aid officers also offer free advice and early care to thousands of acute LALS patients at work, sporting, and public events. In a New Zealand study [106], 96 % of pharmacists recommended RICE (rest, ice, compression, elevation) and saw a mean of nine acute LALS per month. In Australia, pharmacists use a handbook [107] as a resource to guide decision making for acute LALS. The handbook advices RICE, early mobilisation, analgesics, and topical non-steroidal anti-inflammatory drugs (NSAIDs). Possibly, these pharmacists use other forms of knowledge translation such as educational meetings instead of CPGs [108]. It should be noted that four guidelines [36, 38–40] recommended NSAIDs as advised by the pharmacists.

This study is the first systematic review to evaluate the quality of LALS CPGs using the AGREE II tool. The AGREE II consists of 23 items, which are grouped into six domains: scope and purpose (3 items), stakeholder involvement (3 items), rigour of development (8 items), clarity of presentation (3 items), applicability (4 items), and editorial independence (2 items). None of the CPGs scored highly in all domains. The highest domain scores were for domain 4, clarity of presentation with all CPGs scoring above 61%. The second highest domain score was domain 1, scope and purpose with 5 CPGs scoring above 67%. The 2012 Dutch CPG [40] was the only CPG to receive an 100% score in any one domain (domain 4).

The lowest domain score was for domain 5, applicability (discussion of facilitators and barriers to application, provides advice for practical use, consideration of resource implications, and monitoring/auditing criteria) achieving an exceptionally low joint total score of 9% for all CPGs. The five most recent CPGs scored zero for applicability. This is a disturbing finding and further research is needed as to why these CPGs did not address these key components of knowledge translation for clinicians. However, it is probably due to the guidelines being published in peer reviewed journals, the authors being limited by the space available for description of the guideline development process. Conversely, peer review may explain any high scores in editorial independence [40].

Failure to assess whether CPGs are being used correctly is also of concern. Fortunately, this has been assessed independent of the guideline developers. Several papers published by Dutch research groups investigating compliance with an LALS CPG have found moderate compliance by physical therapists in the Netherlands [109, 110]. Recently another Dutch observational study using multi-level analyses of data found discrepancy between the CPGs and practice of the physical therapists [111]. They found that, although not recommended in the CPGs, manual manipulation was applied during treatment in 21% of the patients with functional instability and that patients with acute LALS had only a 38% chance of being treated according to the CPG.

The second lowest domain score was domain 6, editorial independence. Three scored zero for this domain [37, 41, 42]. However, the more recent Dutch CPG [40] scored 97% for editorial independence. This CPG had information under the following headings: Contributors, Funding, Competing Interests, Provenance and Peer Review and Author Affiliations. In this domain of editorial independence, the CPGs are assessed against the following statements:

The views of the funding body have not influenced the content of the CPG.

Competing interests of members of the CPG development group have been recorded and addressed.

The third lowest domain score was for domain 3, rigour of development. Five of the CPGs [36–39, 41] scored below 31% for this domain. Our findings of the lowest scores in these three domains (rigour of development, applicability and editorial independence) are consistent with the findings of a systematic review of CPG appraisal studies [112]. That review showed that despite some increase in quality of CPGs over time, the quality of scores as measured with the AGREE tool has remained moderate to low over the last two decades. They saw significantly lower scores for the same three domains (rigour of development, applicability, and editorial independence) as we did, in CPGs published in 2003 or later.

This systematic review found poor consistency in the reporting of the levels of evidence and strength of acute recommendations. Two CPGs [38, 39] failed to describe the method of assessing the LOE and SOR and the remaining CPGs described differing methods. It is difficult for clinicians and researchers to have confidence in using recommendations if the method of assessment of evidence is not specified or is inconsistent. Current practice shows that CPG developers should use the Grading of Recommendations Assessment, Development and Evaluation (GRADE) tool for assessment of evidence. GRADE is an internationally recognised approach to rate the quality of evidence and the strength of recommendations and is the standard in CPG development [113]. The GRADE handbook states that the strength of recommendation for or against a specific treatment option should be expressed using two categories (weak and strong) [114].

The first CPG assessed in this study was published in 2006, the last in 2013. Factors that might necessitate CPGs to be updated have been discussed in the literature [115, 116]. These include changes in the evidence on the existing benefits and harms of treatments, outcomes considered important, available treatments, evidence that current practice is optimal, values placed on outcomes and resources available for health care. The most frequently recommended time between updates is 2–3 years and the longest is five years. These time periods were found in a systematic review on the guidance for updating CPGs [117]. This indicates that the CPGs published before 2012 are outdated. So four of the CPGs in this study were out of date.

The secondary aim of this study was to determine if CPGs use the same studies to support their recommendations in the acute phase of a LALS. In this systematic review, there is a trend for CPG developers in different disciplines/fields to use the same studies to support their recommendations. However, this secondary objective has not been adequately achieved, in part based on a lack of an appropriate assessment tool. A previous systematic review of CPGs for the physical treatment of osteoarthritis categorised recommendations by grouped treatments with their associated LOE and SOR and then converted these into a scale from − 4 to + 4 [31]. This approach could not be taken in our analysis as the LALS CPGs were smaller in number and two CPGs failed to define LOE and SOR. In addition, many of the acute interventions were recommended on a consensus basis and therefore lacked high quality evidence. In another recent study aimed at critically appraising CPGs for foot and ankle treatments in rheumatoid arthritis, the researchers adopted a descriptive synthesis similar to our method described [118]. All three methods have their limitations and further research is needed to find a more valid and reliable way of assessing the quality of the CPG recommendations.

Inconsistency across CPGs suggests that the most contemporary high-level evidence is not being used by all CPG developers, however this criticism may be tempered by consideration of the differing ages of the CPGs. CPGs can recommend that a treatment be not recommended. For example, in a recent systematic review evaluating treatment strategies for acute LALS the authors found there was insufficient evidence to support the use of ultrasound as an treatment for LALS [119]. This recommendation of not using ultrasound for LALS has appeared in four CPGs [37, 40, 41].

When comparing the recommendations for treatments between American and Dutch guidelines there seems to be disparity. Graded joint mobilisations or mobilisation with movement, pulsating shortwave diathermy, electrotherapy, and low-level laser are not recommended by the Dutch CPGs. However, they are recommended by the American physical therapy CPG, despite some common studies used in development of both CPGs (see Table 3). The interpretation of the evidence between the two CPG development groups is clearly not consistent. Further research and robust studies into the conflicting recommendations are needed.

Only one CPG included a warning about using ice when sleeping with the term “do not” in bold. [36] The specific term “ice burn” was not used. The physical therapy curriculum in Australia advises such a warning [120], specifically, “If you feel any extreme discomfort or pain you must immediately tell …. [your physical therapist]: otherwise, you may be in danger of an ice burn.” As circulation and nerve function may be compromised, caution is also advised when adding compression to cryotherapy [121]. Three CPGs advised against using heat if swelling was present in the acute LALS [38, 39, 41]. This is consistent with other research. Houghton et al. [121] advises that heat is not recommended for tissues inflamed as result of acute injury or exacerbation of chronic inflammatory condition or areas of severe swelling.

Two CPGs [36, 39] written for nurses have recommend, as a component of acute treatment early range-of-motion exercises including foot circles both clockwise and anti-clockwise within 24–48 h of injury or to instruct the client to draw letters of the alphabet with their big toe held in the air. The same two CPGs advised warning for deep venous thrombosis (DVT) risk related to immobilisation for acute LALS [36, 39]. These CPGs may reflect the importance of preventing serious complications (DVT) in the nursing curriculum. However, there seems to be an inadequate understanding of the importance of not overstretching healing ligaments or delaying healing in a severe acute LALS. Further research into the nursing curriculum is recommended to clarify these concepts.

This systematic review found consistency in the use of progressive weightbearing with support in the acute phase for LALS except in two of the CPGs [36, 39]. In the American physical therapist CPG [42] the authors discuss that immobilisation and suturing is associated with improved mechanical stability on stress radiography. In addition, the authors discuss a cadaver study [122] that determined the optimal position for immobilization of severe LALS is a range of dorsiflexion angles between 5 and 15 degrees which reduced anterior talocrural subluxation. This reinforces to the authors that, in severe LALS, mobilisation with alphabet and foot circle exercises should be avoided early in treatment.

## Conclusions

This study highlights areas of deficiency and where improvements are needed in the formulation of future LALS CPGs. The weakest areas were in rigour of development, applicability, and editorial independence. The methodology for assessing recommendations is not consistent between CPG developers. It is a critical question for clinicians whether CPGs are based on high quality evidence. It is of the opinion of the authors of this study that CPG development groups should utilise a validated methodolgy such as GRADE. This study may also inform methodology of critical appraisal of descriptive synthesis of the recommendations of CPGs for other injuries and conditions.

## Limitations

The search procedure included databases, wide search terms and hand searching. Also, the search excluded CPGs not in English, a language filter was applied (see Additional file 1 Search Strategy and List of Articles). Future CPG LALS systematic reviews with international researchers may find CPGs in other languages. The AGREE II tool has limitations in that an absolute rate of agreement between the three reviewers cannot be calculated.

## Additional file

Additional file 1: Search Strategy and List of Articles. (XLSX 23 kb)

## Data Availability

All data generated or analysed during this study are included in this published article and its supplementary information files.

## Abbreviations

AGREE: Appraisal of Guidelines for REsearch and Evaluation
CAI: Chronic ankle instability
CPGs: Clinical practice guidelines
DVT: Deep venous thrombus
EBP: Evidence-based practice
GRADE: Grading of recommendations assessment, development, and evaluation
LALS: Lateral ankle ligament sprains
LOE: Level of Evidence
NSAIDs: Non-steroidal anti-inflammatory drugs
PTOA: Post traumatic ankle osteoarthritis
RCTs: Randomised controlled trials
SOR: Strength of Recommendation
